# Genome-Wide Association Study and QTL Mapping Reveal Genomic Loci Associated with *Fusarium* Ear Rot Resistance in Tropical Maize Germplasm

**DOI:** 10.1534/g3.116.034561

**Published:** 2016-10-13

**Authors:** Jiafa Chen, Rosemary Shrestha, Junqiang Ding, Hongjian Zheng, Chunhua Mu, Jianyu Wu, George Mahuku

**Affiliations:** * College of Agronomy, Synergetic Innovation Center of Henan Grain Crops and National Key Laboratory of Wheat and Maize Crop Science, Henan Agricultural University, Zhengzhou 450002, China; † International Maize and Wheat Improvement Center, 06600 Mexico Distrito Federal, Mexico; ‡ Crop Breeding and Cultivation Research Institute, Shanghai Academy of Agricultural Sciences, Shangai 201403 China; § Maize Research Institute, Shandong Academy of Agricultural Sciences, Jinan 250100, China; ** College of Life Sciences, Henan Agricultural University, Zhengzhou 450002, China; †† International Institute of Tropical Agriculture, 34441 Dar es Salaam, Tanzania

**Keywords:** *Fusarium verticillioides*, maize, association analysis, quantitative trait, disease resistance, GenPred, Shared Data Resources, Genomic Selection

## Abstract

*Fusarium* ear rot (FER) incited by *Fusarium verticillioides* is a major disease of maize that reduces grain quality globally. Host resistance is the most suitable strategy for managing the disease. We report the results of genome-wide association study (GWAS) to detect alleles associated with increased resistance to FER in a set of 818 tropical maize inbred lines evaluated in three environments. Association tests performed using 43,424 single-nucleotide polymorphic (SNPs) markers identified 45 SNPs and 15 haplotypes that were significantly associated with FER resistance. Each associated SNP locus had relatively small additive effects on disease resistance and accounted for 1–4% of trait variation. These SNPs and haplotypes were located within or adjacent to 38 candidate genes, 21 of which were candidate genes associated with plant tolerance to stresses, including disease resistance. Linkage mapping in four biparental populations to validate GWAS results identified 15 quantitative trait loci (QTL) associated with *F. verticillioides* resistance. Integration of GWAS and QTL to the maize physical map showed eight colocated loci on chromosomes 2, 3, 4, 5, 9, and 10. QTL on chromosomes 2 and 9 are new. These results reveal that FER resistance is a complex trait that is conditioned by multiple genes with minor effects. The value of selection on identified markers for improving FER resistance is limited; rather, selection to combine small effect resistance alleles combined with genomic selection for polygenic background for both the target and general adaptation traits might be fruitful for increasing FER resistance in maize.

*Fusarium* ear rot (FER) is one of the most important food and feed safety challenges in maize production worldwide (Munkvold and Desjardins 1997). Apart from reducing the quantity and quality of harvested maize, some of the *Fusarium* spp. produce mycotoxins, which are harmful, and can be fatal to humans and animals consuming contaminated grain (Missmer *et al.* 2006). More than 10 *Fusarium* spp. can cause ear rot, but the two most important are *Fusarium verticillioides* [synonym *F. moniliforme* Sheldon] inciting FER and *F. graminearum* that causes *Gibberella* ear rot (Seifert *et al.* 2003; Mesterházy *et al.* 2012; Kebebe *et al.* 2014). *Fusarium verticillioides* is more prevalent in low rainfall, high humidity environments, common in tropical and subtropical maize production environments, while *F. graminearum* is predominant in cooler, high rainfall maize growing environments (Munkvold 2003). Infection by *F. verticillioides* can result in decreased grain yields, poor grain quality, and contamination by the mycotoxin fumonisin, a suspected carcinogen associated with various diseases in livestock and humans (Munkvold and Desjardins 1997; Fandohan *et al.* 2003; Munkvold 2003; Presello *et al.* 2008).

*Fusarium verticillioides* can survive in soil, healthy seed, and plant residue, and infection of maize can be initiated from seedborne or airborne inoculum as well as systemic infection from the soil through roots to kernels (Morales-Rodríguez *et al.* 2007). Because of the high rate of maize production for subsistence in many developing countries, the solution to the problems of FER and fumonisin contamination is not to strengthen regulations, but rather to reduce fungal infection and mycotoxin levels in grain. The best strategy for controlling FER and reducing incidence of fumonisin contamination is the development and deployment of maize varieties with genetic resistance. Preharvest host resistance is economical to famers, leaves no harmful residue in food or the environment, and is compatible with other control measures. This strategy requires a clear understanding of the genetics of resistance, and the identification of alleles significantly contributing to reduced *F. verticillioides* infection and colonization, and fumonisin production (Mukanga *et al.* 2010).

Resistance to FER is quantitatively inherited and additive, dominant, and additive by dominant effects are important (Boling and Grogan 1965). Mapping studies using biparental populations have shown that resistance to FER is controlled by minor genes with relatively small effects that vary between environments and are not consistent between populations (Mesterházy *et al.* 2012). Robertson-Hoyt *et al.* (2006) and Bolduan *et al.* (2009) reported genotypic correlations between FER resistance and fumonisin accumulation of 0.87 in North Carolina and 0.92 in Germany, respectively, indicating that visual selection of FER resistance should be effective in simultaneously reducing fumonisin contamination. Although genetic variation for resistance to FER exists among maize inbred lines and hybrids, there is no evidence of complete resistance to either FER or fumonisin contamination in maize (Clements and Kleinschmidt 2003; Clements *et al.* 2004). The search for novel resistance genes against *F. verticillioides* is a very important activity in the quest to find a lasting solution to FER problems in maize production. Identification of specific allelic variants that confer improved resistance would permit maize breeders to select for recombinant chromosomes in backcross progeny that have desired target resistance allele sequences in coupling phase with the favorable elite polygenic background, facilitating the improvement of disease resistance without decreasing agronomic performance.

Several studies have identified quantitative trait loci (QTL) associated with resistance to *F. verticillioides* and subsequent reduced fumonisin accumulation (Robertson-Hoyt *et al.* 2006; Bolduan *et al.* 2009). For example, linkage-based mapping studies using F_2:3_ populations derived from two resistant parents and a common susceptible parent identified nine and seven QTL associated with *F. verticillioides* resistance, and three of the QTL were common across the two populations (Pérez-Brito *et al.* 2001). In another study with two populations sharing a common resistant parent, a common QTL was detected on chromosome 4; this QTL was validated in an independent near isogenic line population (Li *et al.* 2011; Chen *et al.* 2012). Other QTL mapping studies have also revealed many QTL for *F. verticillioides* resistance that are stable across environments (Robertson-Hoyt *et al.* 2006; Ding *et al.* 2008). Using the GWAS method, seven SNPs were identified for FER resistance based on a diverse inbred line population comprised of 1687 maize inbred lines (Zila *et al.* 2013, 2014). These studies revealed the presence of genetic variation for FER and the potential for identifying and deploying molecular markers for improving FER resistance in maize.

GWAS has shown great potential for detecting QTL with high resolution in diverse germplasm (Buntjer *et al.* 2005). In *Arabdopsis thaliana*, GWAS was conducted using 213,497 SNPs and 473 accessions to reveal climate-sensitive quantitative trait loci (Li *et al.* 2010). In maize, GWAS has successfully been used to identify several casual genomic loci for different traits (Weng *et al.* 2011; Wang *et al.* 2012b; Liu *et al.* 2014; Samayoa *et al.* 2015). However, GWAS also has shortcomings, such as detection of false positives due to presence of population structure; fortunately, several advanced statistical methods have been developed to reduce the false positive rate (Andersen *et al.* 2005; Yu *et al.* 2006a; Larsson *et al.* 2013). Compared to traditional linkage-based analyses, association mapping offers higher mapping resolution while eliminating the time and cost associated with developing synthetic mapping populations (Flint-Garcia *et al.* 2005; Yu *et al.* 2006b). On the other hand, linkage mapping generates low rates of false positive results, which offset the limitation of so few alleles in offspring populations (Jiang and Zeng 1995; Ding *et al.* 2015b). Combining GWAS and linkage mapping could exploit the complementary strengths of both approaches to identify casual loci (Fulker *et al.* 1999; Pedergnana *et al.* 2014; Motte *et al.* 2014).

In this study, we used GWAS to identify genomic regions associated with FER resistance in tropical maize germplasm populations that were evaluated across three environments in Mexico. GWAS-identified genomic regions were validated through linkage mapping using four biparental populations. Furthermore, we identified a set of tropical maize inbred lines with high levels of FER resistance that can be used to improve FER in maize breeding programs.

## Materials and Methods

### Germplasm materials and experimental design

A collection of 940 elite tropical maize inbred lines was assembled from International Maize and Wheat Improvement Center (CIMMYT) maize breeding programs located in Zimbabwe, Kenya, Colombia, and Mexico, and from the physiology, pathology, and entomology programs was evaluated for disease resistance (Semagn *et al.* 2012; Wen *et al.* 2011**)**. One elite maize inbred line, CML155, was used as a resistant check. This line had previously been identified as highly resistant to FER following multiple years of visual evaluation under field conditions in CIMMYT’s experimental station of Agua Fria (AF), Mexico. Four biparental-derived populations that included a doubled haploid (DH) population composed of 201 lines derived from crossing CML495 (resistant) to LA POSTA SEQ. C7 F64-2-6-2-2-B-B-B (susceptible), designated POP1 and F_2:3_ biparental populations developed from three resistant parents (CML492, CML495, and CML449) crossed to a single susceptible parent (LPSMT), and named POP2 (277 families), POP3 (268 families), and POP4 (272 families), respectively, were evaluated for resistance to FER (Supplemental Material, Table S1).

The GWAS panel of 940 inbred maize lines was screened for FER resistance in two locations: CIMMYT’s experimental station of AF, located in the state of Puebla in Mexico [longitude 97°38′W; latitude 20°28′N; elevation 100–110 masl (meters above sea level)] in 2010 and 2011 (AF10 and AF11); and CIMMYT’s experimental station of Tlaltizapan (TL) located in the state of Morelos, Mexico (longitude 99°7′W; latitude 18°41′N; elevation 940 masl) in 2011 (TL11). Entries were divided into four sets on the basis of maturity. Sets were randomized within the field and each set was blocked using an α-lattice design and replicated three times. Twenty seeds were planted in 2-m row plots, with 0.2 m between plants in a row and 0.75 m between rows. Two seeds were planted per hill and later thinned to a single plant to give a total of 10 plants per plot.

### FER inoculations and evaluation

The experiments were artificially inoculated with a local toxigenic *F. verticillioides* isolate using the nail punch/sponge technique (Drepper and Renfro 1990), ∼7 d after flowering. A single-spore isolate of *F. verticillioides* was increased on sterile maize kernels, incubated for 14 d at 25°. After incubation, the spores were harvested, and concentration estimated using a hemocytometer and adjusted to 5 × 10^6^ spores ml^−1^ in sterile distilled water with 0.2 ml/l Tween-20 surfactant (poly-oxyethylene 20-sorbitan monolaurate). The primary ear of each plant in a plot was inoculated using a nail punch/sponge inoculation method with a suspension that contained 5 × 10^6^ spores ml^−1^ about 7 d after flowering. The same inoculation method was used for both the GWAS panel and QTL mapping population.

At maturity, inoculated ears from each plot were harvested by hand and individually rated for FER symptoms using a seven-point scale, where 1 = no visible disease symptoms, 2 = 1–3%, 3 = 4–10%, 4 = 11–25%, 5 = 26–50%, 6 = 51–75%, and 7 = 76–100% of kernels exhibiting visual symptoms of infection (Reid *et al.* 1995). The overall response of each line, defined as percentage of infected area (PIA) was calculated using the formula described by Pérez-Brito *et al.* (2001). The average FER severity score of each line was named EarRot1-7. During harvesting, another variable, ear rot aspect (ERAspect), was assessed on a per plot basis using a 1–5 scoring scale; where 1 = no visible disease symptoms on kernels, 2 = 1–10%, 3 = 11–20%, 4 = 21–30%, and 5 = 31% or more of the kernels infected (Drepper and Renfro 1990). ERAspect is an assessment of overall cleanliness of the cob (presence or absence of general ear rot symptoms). Other variables evaluated included maturity measures as days to anthesis (DTA) and silking (DTS), plant height, ear height, bad husk cover, and stem lodging. Bad husk cover was rated on a 1–5 scale, where 1 represents husks tightly arranged and extending beyond the ear tip (very good husk cover) and 5 = ear tips exposed (bad husk cover).

### Genotypic data

Total DNA was extracted from young leaves using the cetyltrimethylammonium bromide method (CIMMYT 2005), and DNA quality, purity and quantity for each sample was checked using gel-electrophoresis and spectrophotometer (NanoDrop ND8000, Thermo Scientific). A total of 854 maize inbred lines with good quality DNA were genotyped using an Illumina MaizeSNP50 BeadChip which contained 56,110 SNP markers (Ganal *et al.* 2011). The SNP genotyping was performed on an Illumina Infinium SNP genotyping platform at Cornell University Life Sciences Core Laboratories Center using the protocol developed by the Illumina Company. The genotypic data summary (allele frequency, heterozygous rate, and missing rate) were calculated by PLINK v1.07 software (Purcell *et al.* 2007).

The four biparental populations used for linkage mapping were genotyped by low density markers from the Kompetitive Allele Specific PCR (KASP) genotyping system of LGC Company (http://www.lgcgroup.com/) (Semagn *et al.* 2014). A total of 1250 SNPs were screened to identify markers polymorphic between the two parental lines. Of the polymorphic SNP markers, 200 were selected and used to genotype the entire population. Markers with allele frequency between 0.4 and 0.6 for both DH and F2:3 populations were included in the analysis.

### Statistical analyses

Descriptive statistics (such as mean, range, skewness, and kurtosis) and correlations of phenotypic data were conducted in Excel 2010. Genetic correlation, and best linear unbiased estimates (BLUEs) were calculated using SAS (SAS Institute 2011) with multiple environments traits analysis package (META) which can be found on CIMMYT Dataverse (http://hdl.handle.net/11529/10217) (Vargas *et al.* 2013). For the single environment BLUE, a mixed linear model was performed including line as a fixed effect, days to silking as a fixed linear covariate, and replication and block within replication as random effects. In the combined experimental analysis, each combination of location and year was considered an environment, with a mixed linear model including line as a fixed effect, days to silking (DTS) as a fixed linear covariate, and year, line × environment interaction, replication within environment, and block within replication as random effects.

The ANOVA was conducted in R software with ANOVA (lm) function (R Core Team 2015); the model for ANOVA was as follows:

Single environment ANOVA: Pheno∼Rep+Block:Rep+Entry

Multi environment ANOVA: Pheno∼Env∗Entry+Rep:Env+Block:(Rep:Env)

where *Pheno* was phenotypic data; *Env* was environments, which was the combination of location and year; *Rep* was replication; *Block* was block in α-lattice design; *Entry* was the inbred lines used in this study.

Variance components were estimated using VarCorr function after fitting the linear mixed model (lmer) with the REML option in R software (R Core Team 2015). The single environment repeatability (*H*^2^) was estimated using the following formula (Knapp *et al.* 1985):

$$H^{2}=\sigma_{\text{G}}^{\text{2}}/\left(\sigma_{\text{G}}^{\text{2}}+\sigma_{\text{e}}^{2}/\text{r}\right),$$

Broad-sense heritability (*H*^2^) was estimated using the formula below (Knapp *et al.* 1985):

$$H^{2}=\sigma_{\text{G}}^{\text{2}}/\left(\sigma_{\text{G}}^{\text{2}}+\sigma_{\text{GE}}^{\text{2}}/\text{E}+\sigma_{\text{e}}^{\text{2}}/\text{lr}\right),$$

where σ^2^_G_ is genetic variance, σ^2^_GE_ is genotype × environment interactions variance, σ_e_^2^ is error variance, *E* is the number of environments, and r is the number of replications in each environment.

### Association analysis

A subset of 2000 SNP markers were randomly selected from 10,736 SNPs that remained after removing SNPs with missing values >10%; minor allele frequency of <10%; and physical position interval <50 kb. This subset of SNP markers was used for STRUCTURE analysis (Yu *et al.* 2009). The population structure was determined using an admixture model with correlated allele frequency in software STRUCTURE v2.3.3 (Pritchard *et al.* 2000). A burn-in of 10,000 iterations followed by 100,000 Monte Carlo Markov Chain replicates was conducted to test **k** values (number of subpopulations) in the range of 2–9. Each **k** was replicated four times, and most lines were assigned into clusters with a probability >0.6 (Falush *et al.* 2003).

Principal Component Analysis (PCA) was conducted in Eigensoft V3.0 software (Price *et al.* 2006; Patterson *et al.* 2006). Genetic distance-based neighbor-joining (NJ) analysis and a genetic kinship matrix were conducted using TASSEL V3 (Bradbury *et al.* 2007) and the tree visualized using FigTree v1.3.1 (Rambaut and Drummond 2009). Linkage disequilibrium (LD) measured as *D*′ was calculated using TASSEL software (Bradbury *et al.* 2007). Haplotype was built using the LD-based method as described by Gabriel *et al.* (2002), and SNPs are considered to be in the same haplotype or in “strong LD” if the one-side upper 95% confidence bound on *D*′ was >0.98 and the lower bound was above 0.7, and was calculated using PLINK v1.07 software (Purcell *et al.* 2007).

A mixed linear model that included BLUEs, marker, kinship matrix (*K*), and PCA was conducted using TASSEL software (Bradbury *et al.* 2007). Haplotype generated by PLINK and haplotype genotypes were used to conduct association mapping using the mixed linear model with PCA and Kinship in TASSEL software.

### QTL mapping in biparental populations

Linkage maps were constructed using IciMapping v3.2 with Kosambi method for map distance calculation (Kosambi 1944; Wang *et al.* 2012a). The total map length for POP1 (DH population) was 1260 cM and included 166 SNPs and the average marker interval was 8.83 cM; the map length of POP2 was 991 cM and included 154 SNPs and the average marker interval was 8.93 cM. Linkage maps were not constructed for POP3 and POP4 as the number of retained markers was small (118 for POP3 and 93 for POP4). The Inclusive Composite Interval Mapping (ICIM) method in IciMapping v3.2 was used for QTL mapping (Li *et al.* 2008; Wang *et al.* 2012a). ICIM retains all the advantages of composite interval mapping (CIM) over interval mapping and avoids the possible increase of sampling variance and the complicated background marker selection process that are in CIM (Li *et al.* 2007, 2008). The step of ICIM was set to 1 cM, and the LOD threshold was set to 2.5. The total proportion of phenotypic variance explained by the detected QTL was calculated by fitting all significant SNPs simultaneously in a linear model to obtain *R*^2^_adj_. The proportion of the genotypic variance explained by all QTL was calculated as the ratio of *pG* = *R*^2^_adj_/*h*^2^ (Gowda *et al.* 2015). The Single Marker Analysis (SMA) method in IciMapping V3.1 software was used for POP3 and POP4 QTL mapping, since the number of polymorphic markers were not enough for linkage map constriction. BioMercator V3.0 software (Arcade *et al.* 2004) was used to integrate significant markers to the maize physical map of the B73 reference genome (B73 RefGen_v1). The physical positions and sequence of SNP markers were obtained from the Illumina public ftp site (ftp://ussd-ftp.illumina.com/Whole%20Genome%20Genotyping%20Files/Archived_non-Human_Products/Maize_SNP50/).

Based on GWAS results, the sequences flanking SNP markers significantly associated with FER resistance were used to perform BLAST searches against the “B73” RefGen_v2 (MGSC) (http://blast.maizegdb.org/home.php?a=BLAST_UI) to obtain the physical position of significant SNPs.

### Data availability

The original genotype and phenotype of the GWAS population are available in File S1 and File S2 and the original data of the four biparental populations are available in File S3 (POP1), File S4 (POP2), File S5 (POP3), and File S6 (POP4).

## Results

### Phenotypic data analysis of GWAS panel

Significant phenotypic variation for FER was observed in both the Agua Fria and Tlaltizapan experiments. The mean ear rot severity ranged from 0 to 87% with an overall mean of 22.96% in TL11, from 0 to 47% with an overall mean of 7.76% in AF11, and from 0 to 61% with an overall mean of 9.6% in AF10. Disease severity was higher in Tlatizapan than Agua Fria, possibly revealing differences in aggressiveness of *F. verticilioides* strains used. In the combined analysis, mean ear rot ranged from 0 to 74% with an overall mean of 16.03% (Table 1). The distribution of FER scoring in individual and combined environments was close to normal with a skew toward the lower level of infection (Figure S1). Reflect kurtosis analysis revealed that ear rot resistance was continuously distributed, revealing the quantitative nature of *F. verticillioides* resistance (Figure S1 and Table 1). Both genotypic components of variance (σ^2^_G_) and genotype × environment interaction (σ^2^_GE_) were significant (*P* < 0.01), from the combined ANOVA analysis, and σ^2^_G_ was also significant in the three single environments analysis. The repeatability (*H*^2^) of FER scores was generally high, ranging from 0.89 in TL11 to 0.71 and 0.68 in AF11 and AF10, respectively. In combined analysis, the broad-sense heritability (*H*^2^) of the trials was 0.66, indicating that *F. verticillioides* resistance was controlled by genetic factors and that the data could confidently be used for accurate mapping of *F. verticillioides* resistance genes.

**Table 1:** Descriptive statistics and correlation of PIA parameter for FER resistance for the GWAS panel

Env	Mean (%)	Range (%)	SD	CV (%)	Skewness	Kurtosis	*H*^2^	Correlation			σ^2^_G_	σ^2^_GE_
TL11	22.96	0–87	21.4	93.2	1.1875	0.6274	0.89	1	0.64**	0.34**	0.040**	—
AF11	7.76	0–47	9.49	122.3	2.4636	6.8599	0.71	0.54**	1	0.58**	0.016**	—
AF10	9.59	0–61	8.99	93.7	1.9646	5.1865	0.68	0.26**	0.44**	1	0.005**	—
Combine	16.03	0–74	12.1	75.5	1.2374	1.3768	0.66	0.88**	0.79**	0.59**	0.014**	0.015**

Correlation below the diagonal is phenotypic correlation coefficient; correlation above the diagonal is genotypic correlation coefficient. Env, environments; SD, standard deviation; CV, coefficient of variation; σ^2^_G_, genetic variance; σ^2^_GE_, genotype–environment interactions variance.

** Significant at *P* = 0.01.

Genetic and phenotypic correlation between ear rot aspect, ear rot score, and PIA were significant, ranging from *r* = 0.90 to 0.98 (Table S2). Therefore, subsequent data and GWAS analyses were conducted using PIA as a FER parameter. Genetic and phenotypic correlations between environments were highly significant, and the phenotypic correlation between combined mean and mean of the three single environments was significant (Table 1). Low but significant correlations were observed between FER (PIA) and DTS (Table 2). However, a moderate genetic correlation (*r* = 0.38) was observed between FER resistance and stem lodging. This is expected as *F. verticillioides* can grow within the maize plant as an endophyte, and can become pathogenic and incite stalk rot when conditions become stressful to the plant.

**Table 2:** Phenotypic (below the diagonal) and genetic (above the diagonal) correlation coefficient between FER resistance and agronomic traits

Variable	Ear Rot (PIA)	DTA	DTS	Plant Height	Ear Height	Stem Lodging	Bad Husk Cover
Ear rot (PIA)	1	−0.07*	−0.10**	−0.13*	−0.11*	0.38**	−0.03
DTA	−0.06	1	0.97**	0.25**	0.28**	−0.20**	−0.36**
DTS	−0.08*	0.92**	1	0.28**	0.29**	−0.29**	−0.34**
Plant height	−0.11**	0.24**	0.25**	1	0.83**	0.13**	−0.23**
Ear height	−0.10**	0.24**	0.23**	0.82**	1	0.07*	−0.22**
Stem lodging	0.01	−0.01	−0.007	0.01	0.02	1	0.43**
Bad husk cover	0.00	−0.29**	−0.29**	−0.21**	−0.19**	0.02	1

DTA, days to anthesis; DTS, days to silking.

* Significant at *P* = 0.05.

** Significant at *P* = 0.01.

Response of 940 maize inbred lines to FER revealed several lines that consistently had mean disease severity scores <5% across the three environments. Analysis of combined phenotypic data from the different environments identified 63 maize inbred lines that were highly (PIA <5%) resistant to FER (Table S3). These tropical inbred lines can immediately be used as a source of FER resistance in breeding programs.

### Phenotypic data analysis of QTL mapping populations

Significant phenotypic variation for FER was observed for the four biparental populations (Table S5). For all populations, genotypic components of variance (σ^2^_G_) were significant (*P* < 0.01) from the single environment ANOVA analysis. For combined ANOVA, both genotypic components of variance (σ^2^_G_) and genotype by environment interaction (σ^2^_GE_) were significant (*P* < 0.01) for POP1 and POP2, revealing that *F. verticillioides* populations in the two environments might have been different. In combined analysis, the broad-sense heritability (*H*^2^) of the trials was 0.74 for POP2 and 0.52 for POP1. The repeatability was generally high for each single environment, for example, the repeatability of POP1 in the TL12A environment was 0.73 and in AF12A was 0.69. Those results indicate the data could confidently be used for QTL mapping.

### Genotypic characterization of GWAS panel

**Figure 1:**
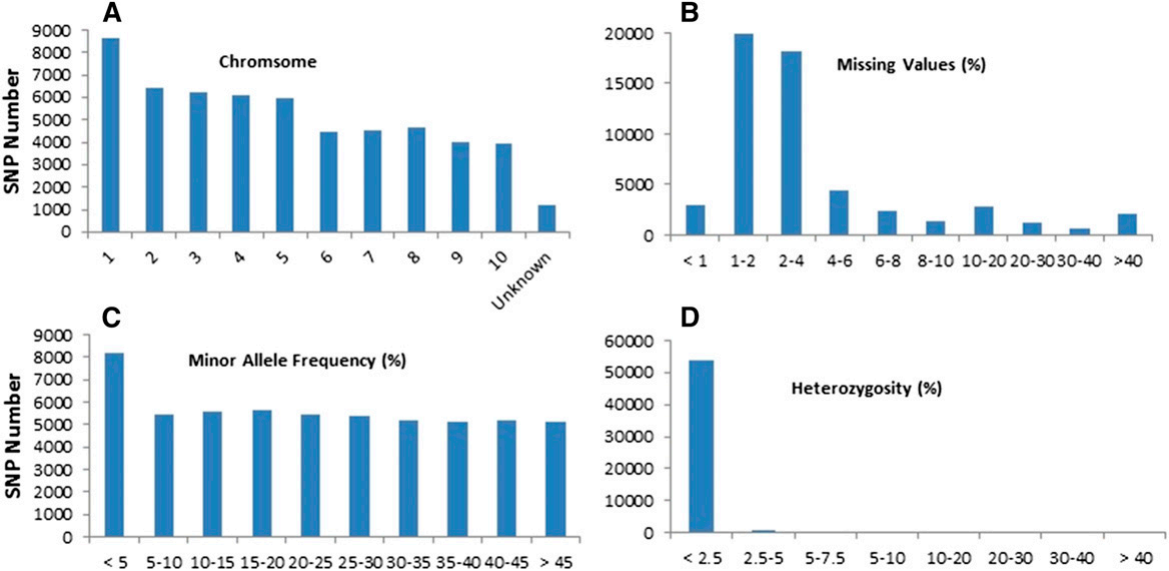
The number of SNP markers per chromosome (A) SNP marker missing value (B) minor allele frequency (C), and marker heterozyosity (D) among 854 maize inbred lines that were genotyped.

A total of 56,110 SNPs were generated for 854 maize inbred lines using the Illumina maize SNP50 BeadChip. The number of SNP markers per chromosome ranged from 3965 SNPs on chromosome 10 to 8625 SNPs on chromosome 1 (Figure 1). The average SNP missing value was 7.0% and 2112 SNPs (3.76%) had a missing value >40%. Of the 56,110 SNPs, 14.6% had a MAF (minor allelic frequency) <0.05, while 55.8% had a MAF >20%. Most of the markers (96.3%) had a heterozygous rate <2.5%, and only 0.01% had a heterozygosity >40%. After eliminating SNP markers with a missing value >40% and MAF <5%, a total of 43,424 SNPs were retained for GWAS.

**Figure 2:**
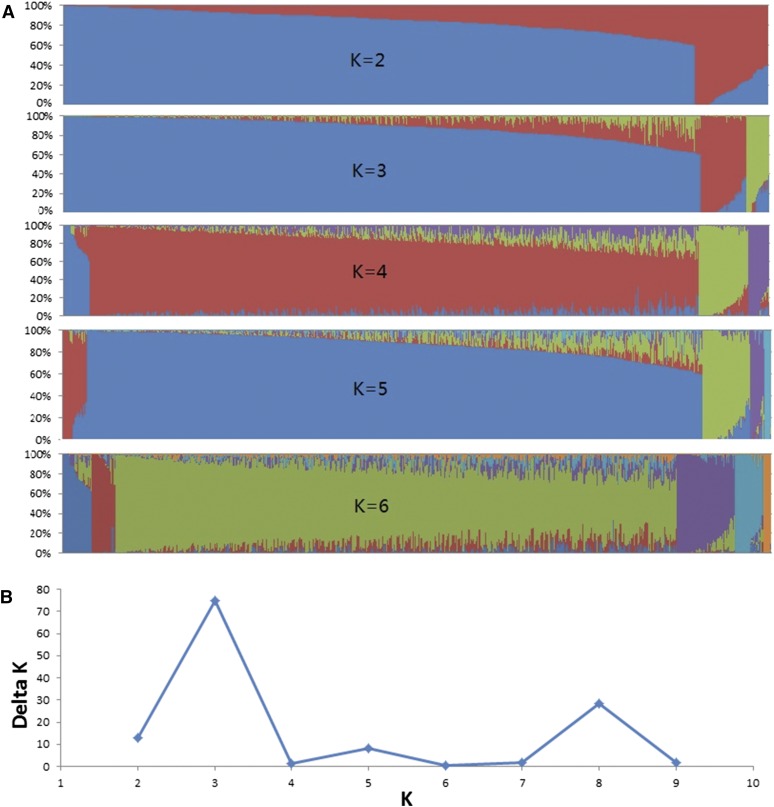
Estimation of number of subpopulations (K) in 818 maize inbred lines used for GWAS analysis using unlinked 2000 random SNP markers. (A) Population structure of maize inbred line panel from *K* = 2 to *K* = 6. The genotype of each line on the figure is represented by a colored line where each color reflects the membership of a cultivar in one of the *K* clusters. (B) Estimation of number of subpopulations (K) in maize inbred line panel using δK values.

From the 940 maize inbred lines evaluated against FER, 818 lines were included in GWAS analysis, after removing lines with >20% heterozygosity and those with >20% missing SNP markers (Figure S2). Population structure estimated using 2000 random SNPs and the software STRUCTURE v2.3.3 divided the inbred lines into three subgroups (Figure 2). Using *k* = 3, 97.3% of the maize inbred lines were assigned to three groups, and only 6.8% of the lines were assigned into mixed population (Figure 2). The largest subgroup (blue color in Figure 2 of the *K* = 3) was composed on germplasm coming from different breeding programs of CIMMYT, including the lowland breeding program, physiology, pathology, and programs in Africa. Most inbred lines in the second subgroup (red color in Figure 2 of *K* = 3) comprised of germplasm derived from CIMMYT’s drought tolerant population LaPostaSeq. The third subgroup (olive green color in Figure 2 of *K* = 3) contained germplasm mainly from CIMMYT’s lowland breeding program. NJ tree constructed using 43,424 SNP markers and 818 maize inbred lines clustered the lines into three major groups (Figure S3), and the grouping was confirmed using PCA analysis (Figure S4). The δK result from STRUCTURE analysis and the absolute difference of eigenvalue between PCs indicted there were three major subgroups in the GWAS panel (Figure 2 and Figure S5). The results obtained following STRUCTUE, PCA, and NJ tree cluster analyses were consistent; therefore, the first three PCAs were used as a covariate in the mixed linear model in GWAS analysis.

### Association mapping for FER resistance

**Figure 3:**
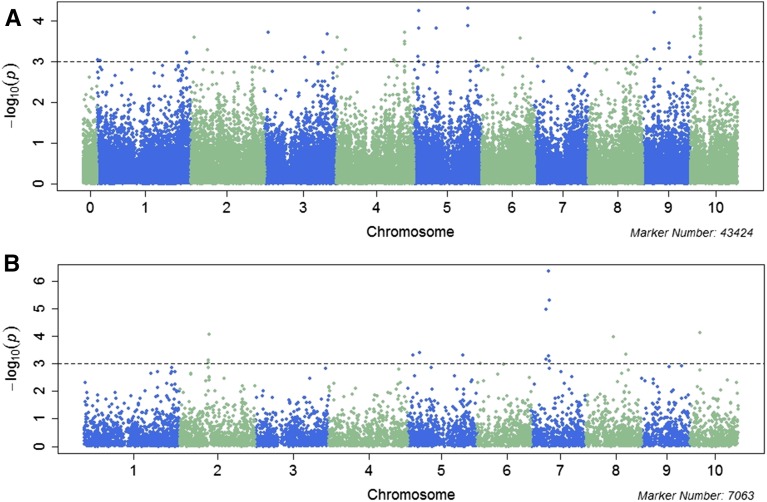
Manhattan plot of genome-wide association analysis (GWAS) for FER resistance with mixed linear model and combined phenotypic data from three environments: (A) single marker GWAS; (B) haplotype-based GWAS. The vertical axis indicates −log_10_ of *P*-value scores, and the horizontal axis indicates chromosomes and physical positions of SNPs.

GWAS analysis using combined phenotypic data identified 45 SNPs that were significantly associated with FER resistance with *P* value <10^−3^ (Figure 3A). The markers were distributed on all chromosomes except chromosome 7; and the number of SNPs per chromosome ranged from 1 on chromosome 8 to 14 on chromosome 10. The most significant SNP was located on chromosome 10 (PZE-110022154) with the lowest *P* value (*P* < 5 × 10^−5^) and it explained 2.06% of the phenotypic variation. The second SNP with lowest *P* value was located on chromosome 5 and it also explained 2.06% of the phenotypic variation. Detailed information of 45 SNPs significantly associated with FER resistance is provided in Table 3. Genome-wide Manhattan plots for single environment analysis are attached (Figure S6). Quantile-quantile plots showed that population structure was controlled well by the mixed linear model (Figure S7).

**Table 3:** SNP and candidate genes significantly associated with FER resistance and detected through single marker GWAS

#*^a^*	SNP	Bin	Position*^b^*	MAF*^c^*	*P* value	*R*^2^	Candidate Genes	SNP Location	Annotation
S1	PUT-163a-16926058-1127	1.00	2,786,055	0.39	9.16E−04	0.014	GRMZM2G041881	3 UTR	Nascent polypeptide-associated complex
S2	PZE-101018023	1.01	10,506,267	0.20	9.64E−04	0.014	GRMZM2G028469	Promoter	—
S3	SYN19964	1.11	285,314,047	0.27	5.97E−04	0.015	GRMZM2G110295	3 UTR	Antifreeze protein
S4	SYN3011	1.11	286,228,712	0.14	6.25E−04	0.015	GRMZM2G178341	3 UTR	Ribosomal protein S13
S5	PZE-102018300	2.02	8,733,661	0.44	2.54E−04	0.018	GRMZM2G443445	Exon	GroES-like
S6	PZE-102073397	2.04	53,583,850	0.13	5.29E−04	0.017	GRMZM2G069093	Promoter	Plant peroxidase
S7	PZE-103018799	3.03	10,791,638	0.26	1.99E−04	0.019	GRMZM2G024551	3 UTR	—
S8	PZE-103079779	3.05	128,563,291	0.13	8.10E−04	0.015	GRMZM2G175968	Promoter	—
S9	SYN24165	3.06	187,947,934	0.26	6.02E−04	0.015	GRMZM2G085392	Exon	Dense granule Gra7 protein
S10	PZE-103149185	3.07	201,056,001	0.29	2.17E−04	0.017	AC207628.4	Intron	IQ calmodulin-binding region
S11	PZE-104001384	4.01	1,497,071	0.22	2.61E−04	0.018	GRMZM2G156346	Promoter	Flagellar motor switch protein
S12	PZE-104025032	4.04	29,025,217	0.39	5.31E−04	0.016	Intergenic	—	—
S13	SYN6472	4.08	183,999,530	0.41	9.27E−04	0.014	GRMZM2G115499	Exon	—
S14	PZE-104130779	4.09	217,656,184	0.33	3.27E−04	0.017	GRMZM2G702806	Exon	2-oxoglutarate (2OG) and Fe(II)-dependent oxygenase superfamily protein
S15	PZE-104130780	4.09	217,656,207	0.33	3.76E−04	0.016	GRMZM2G702806	Exon	2-oxoglutarate (2OG) and Fe(II)-dependent oxygenase superfamily protein
S16	PZE-104130783	4.09	217,656,309	0.33	1.97E−04	0.018	GRMZM2G702806	Exon	2-oxoglutarate (2OG) and Fe(II)-dependent oxygenase superfamily protein
S17	PZE-105024161	5.02	11,879,005	0.21	7.66E−04	0.014	Intergenic	—	—
S18	PZE-105029276	5.02	15,202,871	0.45	5.64E−05	0.021	Intergenic	—	—
S19	PZE-105029277	5.02	15,202,993	0.44	1.57E−04	0.018	Intergenic	—	—
S20	SYN32921	5.03	72,324,287	0.09	1.52E−04	0.021	GRMZM2G029879	Intron	Cyclin-related
S21	PZE-105116484	5.04	172,983,404	0.17	5.06E−05	0.021	GRMZM2G128146	Promoter	Glucose/ribitol dehydrogenase
S22	PZE-105116502	5.04	172,990,198	0.16	1.32E−04	0.018	GRMZM2G128228	Exon	—
S23	PZE-106068510	6.05	121,834,796	0.31	2.69E−04	0.017	GRMZM2G341027	Exon	—
S24	SYN12691	6.07	164,074,687	0.37	8.67E−04	0.014	Intergenic	—	—
S25	PZE-108104835	8.06	158,591,683	0.41	7.58E−04	0.014	GRMZM2G002135	5 UTR	Phospholipid/glycerol acyltransferase family protein
S26	PZE-109011484	9.01	11,972,127	0.14	9.39E−04	0.014	GRMZM2G467169	3 UTR	—
S27	PZE-109031748	9.03	37,162,489	0.23	5.06E−04	0.015	GRMZM2G034318	Promoter	—
S28	PZE-109031963	9.03	37,423,712	0.17	6.24E−05	0.020	Intergenic	—	—
S29	PZE-109050938	9.03	85,677,755	0.24	3.63E−04	0.016	GRMZM2G095206	Exon	Glucose/ribitol dehydrogenase
S30	PZE-109050944	9.03	85,678,508	0.24	4.77E−04	0.016	GRMZM2G095206	5 UTR	Glucose/ribitol dehydrogenase
S31	SYN6661	9.08	150,241,000	0.14	7.86E−04	0.014	GRMZM2G148057	Intron	Kinase interacting (KIP1-like) family protein
S32	PZE-110012997	10.02	11,675,413	0.29	2.50E−04	0.017	GRMZM2G413943	Exon	—
S33	PZE-110022153	10.03	30,829,449	0.10	8.33E−05	0.019	GRMZM2G010669	5 UTR	Transcription factor, MADS-box
S34	PZE-110022154	10.03	30,829,471	0.10	5.00E−05	0.021	GRMZM2G010669	5 UTR	Transcription factor, MADS-box
S35	PZE-110022412	10.03	31,526,825	0.14	6.75E−04	0.014	GRMZM2G560307	Promoter	—
S36	PZE-110022609	10.03	32,154,695	0.14	2.11E−04	0.017	GRMZM2G544512	Promoter	—
S37	PZE-110022613	10.03	32,155,942	0.14	5.81E−04	0.015	Intergenic	—	—
S38	PZE-110022625	10.03	32,159,272	0.14	9.98E−04	0.013	Intergenic	—	—
S39	PZE-110022694	10.03	32,402,406	0.13	1.36E−04	0.018	Intergenic	—	—
S40	PZE-110022708	10.03	32,475,067	0.14	2.00E−04	0.017	Intergenic	—	—
S41	PZE-110022724	10.03	32,493,898	0.14	2.74E−04	0.017	GRMZM2G027431	5 UTR	Putative endonuclease or glycosyl hydrolase
S42	PZE-110022808	10.03	32,797,753	0.15	4.13E−04	0.016	Intergenic	—	—
S43	PZE-110022827	10.03	32,979,981	0.14	1.59E−04	0.018	GRMZM2G109783	Promoter	Protein kinase C
S44	PZE-110022852	10.03	33,120,424	0.13	9.15E−05	0.019	Intergenic	—	—
S45	PZE-110022891	10.03	33,194,481	0.14	6.54E−04	0.015	Intergenic	—	—

a The name used in the software BioMereator V3.0.

b The physical position based on B73 reference genome v1 (B73 RefGen_V1).

c Minor allele frequency.

Haplotype built based on LD as described by Gabriel *et al.* (2002) resulted in 7063 haplotypes (Table S4). The maximum number of markers per haplotype was 19, the minimum 2, and the average number was 2.96 SNPs per haplotype. Haplotype-based GWAS in a mixed linear model identified 15 haplotypes that were significantly associated with FER resistance and these were distributed in bin 2.05, 5.03/5.04, 7.02, 8.03/8.04, and 10.03 (Figure 3B). Haplotype analysis increased the power of marker detection; for example, haplotype 5076 on chromosome/bin 7.02 that was significantly associated with FER resistance (*P* value = 4.45 × 10^−7^) was not detected in single marker GWAS analysis (Table 4). However, some markers were detected by both single marker and haplotype-based GWAS analysis. Haplotype 4168 on chromosome 5 accounted for 3.1% of variation for FER and the two markers PZE-105116484 and PZE-105116502 associated with this haplotype explained 2.1 and 1.8% of phenotypic variation for FER, respectively (Table 4).

**Table 4:** Haplotypes and respective candidate genes that were significantly associated with FER resistance detected through haplotype-based GWAS

#*^a^*	Haplotype	Bin	First marker position*^b^*	End marker position*^b^*	SNPs Number	Alleles Number	*P* value	*R*^2^	Candidate Genes	Annotation
H1	1459	2.05	88,710,768	88,847,068	4	5	8.94E−04	0.027	AC204390.3	—
H2	1460	2.05	89,154,656	89,280,724	8	6	7.50E-04	0.032	GRMZM2G091313	—
H3	1467	2.05	91,759,712	91,845,565	5	5	9.00E-05	0.031	GRMZM2G562083	—
H4	3606	5.02	15,202,871	15,202,993	2	4	5.04E−04	0.023	GRMZM2G100412	Oxidation reduction
H5	3693	5.03	36,846,799	37,030,576	11	6	3.96E−04	0.031	GRMZM2G350853	—
H6	4168	5.04	172,983,404	173,032,965	4	7	4.96E−04	0.031	GRMZM2G128146	Glucose/ribitol dehydrogenase
H7	5049	7.02	45,334,864	45,530,990	4	3	1.09E−05	0.031	GRMZM2G058128	—
H8	5053	7.02	46,245,964	46,406,735	8	9	7.02E−04	0.041	GRMZM2G095557	—
H9	5075	7.02	53,371,838	53,372,042	2	3	5.16E−04	0.020	GRMZM2G023184	DNA topological change
H10	5076	7.02	53,609,623	53,610,328	2	3	4.45E−07	0.039	GRMZM2G513532	—
H11	5080	7.02	55,590,091	55,778,923	4	6	5.13E−06	0.043	GRMZM2G048257	Zinc ion binding
H12	5083	7.02	56,459,593	56,460,086	2	4	8.02E−04	0.022	—	—
H13	5754	8.03	86,545,938	86,546,527	2	4	1.09E−04	0.027	GRMZM2G415172	C5YXL1_SORBI Putative uncharacterized protein Sb09g019530
H14	5923	8.05	125,354,692	125,362,629	2	4	4.49E−04	0.023	AC1**9**7021.3	Zinc finger family protein
H15	6676	10.03	30,829,449	30,829,471	2	2	7.74E−05	0.020	GRMZM2G010669	Transcription factor, MADS-box

a The name used in the software BioMereator V3.0.

b The physical position based on B73 reference genome v1 (B73 RefGen_V1).

### QTL mapping of FER resistance

Five QTL were detected in the DH population (POP1); two on chromosome 1 and one each on chromosomes 2, 3, and 5 (Table 5). The QTL on chromosome 2 accounted for 15.41% of the total phenotypic variation observed for FER in this population; while the QTL on chromosome 5 explained 13.56% of the phenotypic variation (Table 5). Combined, the five QTL detected in POP1 explained 49% of the total phenotypic variance observed for FER. For POP2, an F_2:3_ population, six QTL were detected that together accounted for 25% of the observed phenotypic variation. The QTL on chromosome 1 accounted for 11.36% of the phenotypic variation for FER resistance. The QTL in bin 4.03/04 explained 9.27% of the phenotypic variance, while that in bin 10.03 accounted for 7.82% of the phenotypic variance (Table 5). SMA was used for QTL mapping for POP3 and POP4 as few polymorphic markers were detected in these populations. For POP3, six markers were significantly associated with FER resistance and these were distributed in three regions of chromosome 5; bins 5.03, 5.04, and 5.05 (Table 5). The phenotypic variation for FER explained by these markers ranged from 4.56 to 6.73%, revealing that these were minor QTL. The SNP in bin 5.04 had the greatest effect, explaining 6.73% of the observed phenotypic variance for FER. For POP4, four markers were associated with FER resistance and these were in bin 2.04, 2.06, and 2.07 (Table 5). The phenotypic variation explained by these markers ranged from 12.56 to 15.84% and the SNP in bin 2.07 had the largest effect, explaining 15.84% of the phenotypic variance for FER resistance (Table 5).

**Table 5:** QTL mapping of FER resistance in four biparental populations

Population	Name	Bin	Position	Left Marker	Right Marker	LOD	PVE (%)	Add*^a^*	Dom*^a^*
POP1	Q1	1.04	83	PZA03168_5	PZA01267_3	3.68	5.68	4.55	—
POP1	Q2	1.07	166	PHM5480_17	PHM14614_22	4.77	5.99	−4.68	—
POP1	Q3	2.03/04	56	PZA00590_1	PZA02378_7	11.15	15.41	7.57	—
POP1	Q4	3.06/07	70	PZA03647_1	PHM13673_53	3.62	4.26	3.96	—
POP1	Q5	5.03	56	PHM12992_5	PHM2524_4	10.24	13.56	7.1	—
POP2	Q6	1.03/04	2	PZA02490_1	PZA00240_6	8.08	11.36	6.67	−0.53
POP2	Q7	3.05	54	PZB02179_1	PHM9914_11	4.85	6.11	−4.58	2.15
POP2	Q8	4.03/04	26	PZA02358_1	PHM3112_9	6.53	9.27	−5.83	0.09
POP2	Q9	4.06/08	50	PHM5572_19	PHM14618_11	3.19	3.93	−1.03	5.29
POP2	Q10	9.01/02	8	sh1_12	PHM9374_5	3.47	3.94	3.74	1.12
POP2	Q11	10.03	36	PHM4066_11	PZA03607_1	5.28	7.82	5.45	0.06
POP3	Q12	5.03	32,599,447	PHM4647_8	—	3.06	4.96	0.09	−0.02
POP3	Q13	5.04	164,230,168	PZA00148_3	—	4.19	6.73	0.11	−0.01
POP3	Q13	5.04	166,468,431	PZA02981_2	—	4.1	6.59	0.11	−0.01
POP3	Q13	5.05	179,060,561	PHM1899_157	—	3.08	4.99	0.09	0.02
POP3	Q13	5.05	179,953,106	PZA02633_4	—	2.81	4.56	0.09	0.03
POP3	Q13	5.05	180,603,220	PZA02356_7	—	2.81	4.56	0.09	0.03
POP4	Q14	2.04	40,967,991	PHM10404_8	—	7.95	12.56	3.9	−0.55
POP4	Q15	2.06	166,659,759	PZA03692_1	—	10.2	15.8	4.12	−1.11
POP4	Q15	2.07	176,000,581	PZA00224_4	—	10.22	15.84	4.04	−0.59
POP4	Q15	2.07	194,696,039	PHM793_25	—	9.42	14.69	4	−0.74

Name: indicates the QTL name used in the software BioMereator V3.0. Position: for POP1 and POP2 indicates the genetic position on the linkage map; for POP2 and POP3 indicated the physical position of the marker on B73 reference genome (B73Ref_V1). LOD, logarithm of odds ratio; PVE, phenotypic variance explained; Add, additive effect; Dom, dominance effect.

a A positive value means the favorite allele comes from a resistant parent and negative value means the favorite allele comes from a susceptible parent.

**Figure 4:**
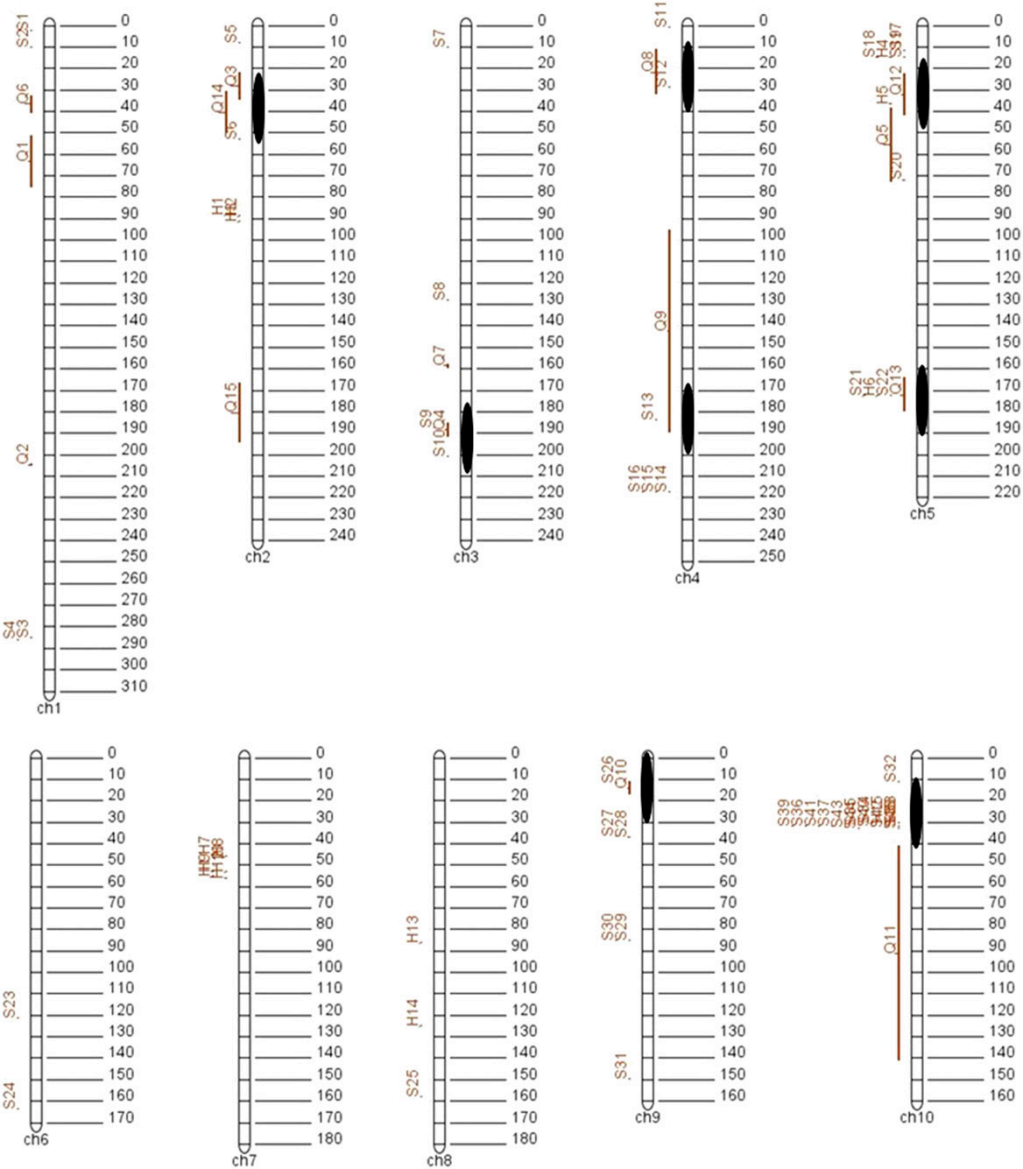
Visualization of all loci associated with FER resistance that were detected in this study using the software BioMereator V3.0. The black ovals represent the location of the eight loci detected by both GWAS and linkage mapping. The numbers on the right of the chromosome indicate the physical position of the chromosome with million base pair as unit.

Forty-five single SNP markers and 15 haplotypes identified through GWAS, together with 15 QTL identified through linkage mapping were integrated onto a maize physical map using the software BioMereator V3.0 (Sosnowski *et al.* 2012). The map generated by the software was convenient for visualizing QTL and significant SNPs together. Eight common loci were identified on six chromosomes; on chromosome/bin 2.04, 3.06, 4.04, 4.08, 5.03, 5.04, 9.01, and 10.03 (Figure 4). The QTL on chromosome 2 in bin 2.04 was detected in two biparental populations as well as single marker GWAS. The chromosome 5 (bin 5.04) locus was detected in one biparental population and by both single marker and haplotype GWAS. The locus on chromosome 10 bin 10.3 contained 14 significant SNP markers, one haplotype and one QTL.

**Figure 5:**
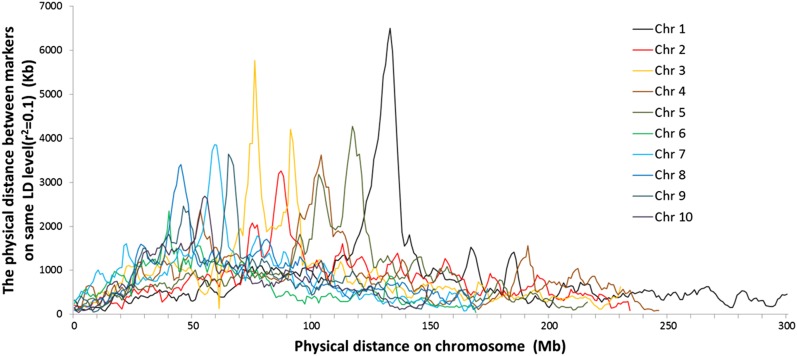
Linkage disequilibrium (LD) decay distance on each of the 10 maize chromosomes for the GWAS panel used in this study.

## Discussion

### Resistance donor

Developing host resistance is the preferred strategy for managing FER, especially for smallholder farmers across the tropics, who largely produce maize for their own consumption, and often lack resources to adopt other control strategies. However, effective use of this strategy requires identification of sources of resistance that are stable and effective across environments. We evaluated 940 maize inbred lines in three environments and identified 63 inbred lines that were highly resistant to *F. verticillioides*. These sources of FER resistance complement a few that have been reported in tropical germplasm (Pérez-Brito *et al.* 2001; Small *et al.* 2012). The broad-sense heritability (*H*^2^ = 0.66) was high, revealing that FER resistance was genetically controlled, thus, significant improvements for FER resistance can be achieved through breeding. Furthermore, the 63 inbred lines resistant to FER constitute a valuable tool for understanding the genetic basis and architecture of FER resistance in tropical maize germplasm. These lines should be evaluated in multiple environments to confirm stability of FER resistance.

### QTL for FER resistance

Forty-five SNPs and 15 haplotypes associated with FER resistance were identified through single marker and haplotype-based GWAS and 15 QTL were identified through linkage mapping in four biparental populations. Using the software BioMereator V3.0, eight loci, containing significant markers from GWAS and linkage mapping were identified (Figure 4). Six loci on chromosomes/bin 3.06, 4.04, 4.08, 5.03, 5.04, and 10.03 are in regions that have previously been reported (Chen *et al.* 2012; Ding *et al.* 2008; Li *et al.* 2011; Pérez-Brito *et al.* 2001; Robertson-Hoyt *et al.* 2006; Zhang *et al.* 2007), while two loci, on chromosomes/bin 2.04 and 9.01 are new loci, identified in this study. Two of the loci on chromosomes 4.04 and 9.01 are in regions containing genes encoding putative proteins of unknown function, while six loci are in regions that have been associated with stress tolerance, including FER resistance. Results from this study concur with previous reports (Boling and Grogan 1965; Zila *et al.* 2014) that FER resistance is a complex trait conditioned by multiple genes with minor effects.

The loci on chromosome 5.04 contained two significant SNPs, one haplotype and a QTL detected through linkage mapping. This chromosome region has previously been reported in three independent QTL mapping studies (Pérez-Brito *et al.* 2001; Robertson-Hoyt *et al.* 2006; Ding *et al.* 2008). Candidate gene analysis revealed that this QTL was in a region containing a putative protein encoding a glucose/ribitol dehydrogenase protein that catalyzes the oxidation of D-glucose to D-β-gluconolactone using NAD or NADP as a coenzyme in the cell development. This gene belongs to a subset of short-chain dehydrogenase and reductase family of genes which are involved in different biochemical processes including pathogen toxin reduction (Meeley *et al.* 1992; Moummou *et al.* 2012).

The locus on the long arm of chromosome 4 (bin 4.08), detected through both GWAS and linkage mapping, has previously been reported (Li *et al.* 2011; Chen *et al.* 2012). Markers within this locus localized to a putative protein of unknown function from maize. However, blastp analysis revealed that it had high homology to Arabidopsis 2OG-Fe (II) oxide reductase, a gene that is involved in regulating giberellic acid and abscisic acid biosynthesis, which are involved in plant tolerance to stress, including disease resistance (van Damme *et al.* 2008; Han and Zhu 2011). Furthermore, chromosome 4.08 is a hot spot region for disease resistance in maize and has been found to harbor resistance QTL to eight maize diseases (Wisser *et al.* 2006). This would be a good target for developing markers to simultaneously introgress multiple disease resistance genes.

The chromosome 10.03 locus containing 13 SNPs and one QTL is located in a region conditioning resistance to multiple maize disease, including rp1 and rp5 that confers resistance to common rust (Wisser *et al.* 2006). The candidate with the lowest *P* value in this region encoded an MADS-box transcription factor (Parenicová *et al.* 2003). MADS-box family genes are involved in controlling major aspects of plant development, including embryo and seed development (Gramzow and Theissen 2010), and may increase seed vigor and subsequently increase tolerance to diseases.

Other important resistance loci identified in this study included chromosomes 3.06 and 5.03. These two loci have previously been reported associated with resistance to FER in two QTL mapping studies (Robertson-Hoyt *et al.* 2006; Ding *et al.* 2008). The locus on bin 3.06 encoded a dense granule Gra7 protein and the bin 5.03 locus was a putative protein of an unknown function. In addition, two new loci were identified, on chromosome 2.04 and 9.01. The chromosome 2.04 locus was associated with a plant peroxidase gene that is involved in cell wall fortification (Kolattukudy *et al.* 1992). The chromosome 9.01 locus encoded a protein of unknown function. Although many SNPs localized to genic regions, the currently limited understanding of pathways contributing to FER resistance restricts our ability to precisely predict what genes might be involved in resistance to this complex disease. However, information from this study provides a basis for further research into elucidating the genetic architecture and pathways leading to FER resistance in maize.

### Haplotype-based GWAS analysis

Because of the rapid LD between markers, haplotype analysis may provide more detection power compared to single marker GWAS and is more practical for breeding (Yan *et al.* 2011). Four methods are commonly used to build haplotypes (Gabriel *et al.* 2002; Yan *et al.* 2009; Gore *et al.* 2009; Lu *et al.* 2010, 2011; Ding *et al.* 2015a): (1) use of a fixed number of markers as a window to slide across the chromosome to build the haplotype; (2) use of a fixed physical distance interval of 10 kb in maize to build the haplotype; (3) use of gene-based physical position to build the haplotype; and (4) use of LD information to put high LD markers together to constitute a haplotype. The marker density in our study was medium to high so we chose the LD-based haplotype build method. Using this approach, haplotype GWAS detected some resistance loci that were not detected by single marker GWAS, whereas the single marker result was reflected in haplotype-based GWAS. This indicates that haplotype-based GWAS has a high marker detection efficiency but requires high density markers to build a haplotype. On-going genotyping by sequencing projects will furnish enough marker density to exploit the advantages of haplotype-based GWAS.

### Candidate genes colocalized with associated SNPs

SNPs and haplotypes associated with FER resistance were located within or adjacent to 38 putative candidate genes which were obtained from the MaizeGDB (http://www.maizegdb.org/) genome browser based on physical position of significant SNPs, MaizeCyc database version 2.0 (http://maizecyc.maizegdb.org/). The Phytozome database (http://phytozome.jgi.doe.gov/pz/portal.html) that was used for defining relevant pathways and annotating possible functions of candidate genes (Caspi *et al.* 2010) could annotate functions to 21 out of the 38 candidate genes (Table 3 and Table 4). Thirteen of the 45 SNPs localized to intergenic regions, 10 were inside exons, nine were located in introns, and nine were located in promoters; five localized to the 3′ untranslated region and five to the 5′ untranslated region (Table 3). The most significant SNP on chromosome/bin 5.04 was in a region associated with a gene encoding a glucose/ribitol dehydrogenase, a protein that catalyzes the oxidation of D-glucose to D-β-gluconolactone using NAD or NADP as a coenzyme. This gene family is a subset of short-chain dehydrogenases and reductases, involved in pathogen toxin reduction (Meeley *et al.* 1992; Moummou *et al.* 2012). These results reveal the complex nature of FER resistance in tropical maize, and indicate that various mechanisms might be involved in conditioning FER resistance, including complex biosynthesis processes, which also might include interactions between multiple metabolic pathways.

### Conclusion

This study identified a set of inbred lines that can potentially be used as sources of resistance to develop hybrids with resistance to FER. Further validation of the potential sources of resistance in multiple environments is required, but the small number of inbred lines makes this process cost-effective. Eight loci harboring FER QTL were identified through integrating GWAS and linkage mapping results. Two are new loci while six colocalized to loci that have previously been described (Chen *et al.* 2012; Ding *et al.* 2008; Li *et al.* 2011; Pérez-Brito *et al.* 2001; Robertson-Hoyt *et al.* 2006; Zhang *et al.* 2007). Some SNPs associated with these loci localized to within or close to genes with known function. Candidate gene analyses for significant SNPs provided targets for further research to elucidate mechanisms of FER resistance. Our results confirmed earlier reports that many genes are involved in FER resistance.

## Supplementary Material

Supplemental Material
