# Supplementary material for: Genome-Wide Association Study and QTL Mapping Reveal Genomic Loci Associated with Fusarium Ear Rot Resistance in Tropical Maize Germplasm
Source: G3 (Bethesda). 2016 Oct 13;6(12):3803–15. doi: 10.1534/g3.116.034561 (PMC5144952; doi:10.1534/g3.116.034561)
Supplement: Supplemental Material [file supp_6_12_3803__index.html]

Genome-Wide Association Study and QTL Mapping Reveal Genomic Loci Associated with Fusarium Ear Rot Resistance in Tropical Maize Germplasm — Supplemental Material 

# Genome-Wide Association Study and QTL Mapping Reveal Genomic Loci Associated with *Fusarium* Ear Rot Resistance in Tropical Maize Germplasm

## Supplemental Material for Chen *et al.*, 2016

**Files in this Data Supplement:**

- Figure S1 - Phenotypic distribution of *Fusarium* ear rot (FER) rating scores of 940 maize inbred lines in different environments. (.pdf, 20 KB)
- Figure S2 - The distribution of heterozygosity and missing value in 854 inbred lines genotyped in this study. (.pdf, 21 KB)
- Figure S3 - Neighbor-joining (NJ) tree for 818 maize inbred lines used for GWAS. (.pdf, 99 KB)
- Figure S4 - Population structure based on principal component analysis (PCA) for 818 maize inbred lines included in association mapping (a: PC1 vs. PC2; b: PC2 vs PC3; c: PC1 vs PC3). (.pdf, 188 KB)
- Figure S5 - Plot of eigenvalues of the principal components showing first three principal components that could explain most of the variance observed for the GWAS panel. (.pdf, 17 KB)
- Figure S6 - Manhattan plots of mixed linear model for *Fusarium* ear rot (FER) resistance for 818 maize inbred lines in single environment GWAS. (.pdf, 250 KB)
- Figure S7 - QQ plots resulting from single markers GWAS for the GWAS panel. (.pdf, 98 KB)
- Table S1 - Type and size of the four bi-parental populations used in this study. (.xlsx, 9 KB)
- Table S2 - Phenotypic (below the diagonal) and genetic (above the diagonal) correlation coefficients between different disease evaluation parameters in GWAS panel. (.xlsx, 9 KB)
- Table S3 - Best-bet *Fusarium* ear rot (FER) resistance donors. (.xlsx, 12 KB)
- Table S4 - Information on the haplotypes and average number of SNPs constituting the haplotype. (.xlsx, 9 KB)
- Table S5 - Population, mean, genotypic variance component and heritability for Fusarium ear rot resistance in four bi-parental populations. (.xlsx, 9 KB)
- File S1 - Genotype data for 818 tropical maize inbred lines constituting the GWAS panel used in this study. (.zip, 10 MB)
- File S2 - Phenotypic data for 818 tropical maize inbred lines used in this study. (.zip, 21 KB)
- File S3 - Genotype and phenotype data for bi-parental doubled haploid (DH) population composed of 201 lines derived from crossing CML495 (resistant) to LA POSTA SEQ. C7 F64-2-6-2-2-B-B-B (susceptible) maize inbred lines and designated POP 1. (.zip, 13 KB)
- File S4 - Genotype and phenotype data of an F2:3 bi-parental populations developed from crossing CML492 (resistant) to LPSMT (susceptible) and composed of 277 families, and designated POP 2. (.zip, 11 KB)
- File S5 - Genotype and phenotype data F2:3 bi-parental populations developed from crossing CML495 (resistant) with LPSMT (susceptible), designated POP3 and composed of 268 families. (.zip, 17 KB)
- File S6 - Genotype and phenotype data for an F2:3 bi-parental populations developed from crossing CML449 (resistant) with LPSMT (susceptible), and designated POP4 and composed of 272 families. (.zip, 15 KB)
