# Supplementary material for: Genome-Wide Association Study and QTL Mapping Reveal Genomic Loci Associated with Fusarium Ear Rot Resistance in Tropical Maize Germplasm
Source: G3 (Bethesda). 2016 Oct 13;6(12):3803–15. doi: 10.1534/g3.116.034561 (PMC5144952; doi:10.1534/g3.116.034561)
Supplement: Supplemental Material [file supp_g3.116.034561_FigureS2.pdf]

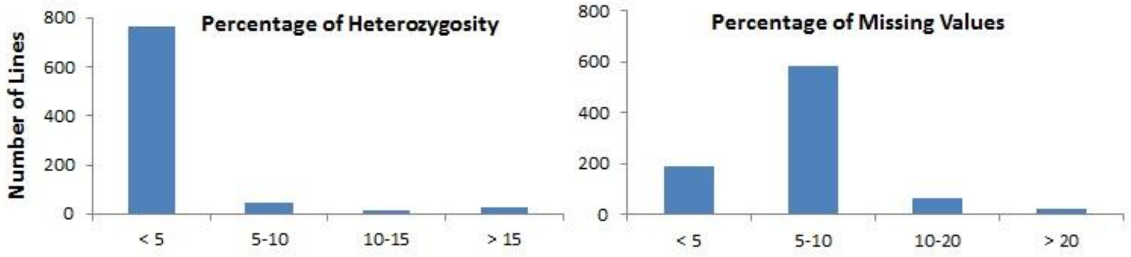

Figure S2. The distribution of heterozygosity and missing value in 854 inbred lines genotyped in this study.
