# Supplementary material for: Genome-Wide Association Study and QTL Mapping Reveal Genomic Loci Associated with Fusarium Ear Rot Resistance in Tropical Maize Germplasm
Source: G3 (Bethesda). 2016 Oct 13;6(12):3803–15. doi: 10.1534/g3.116.034561 (PMC5144952; doi:10.1534/g3.116.034561)
Supplement: Supplemental Material [file supp_g3.116.034561_FigureS3.pdf]

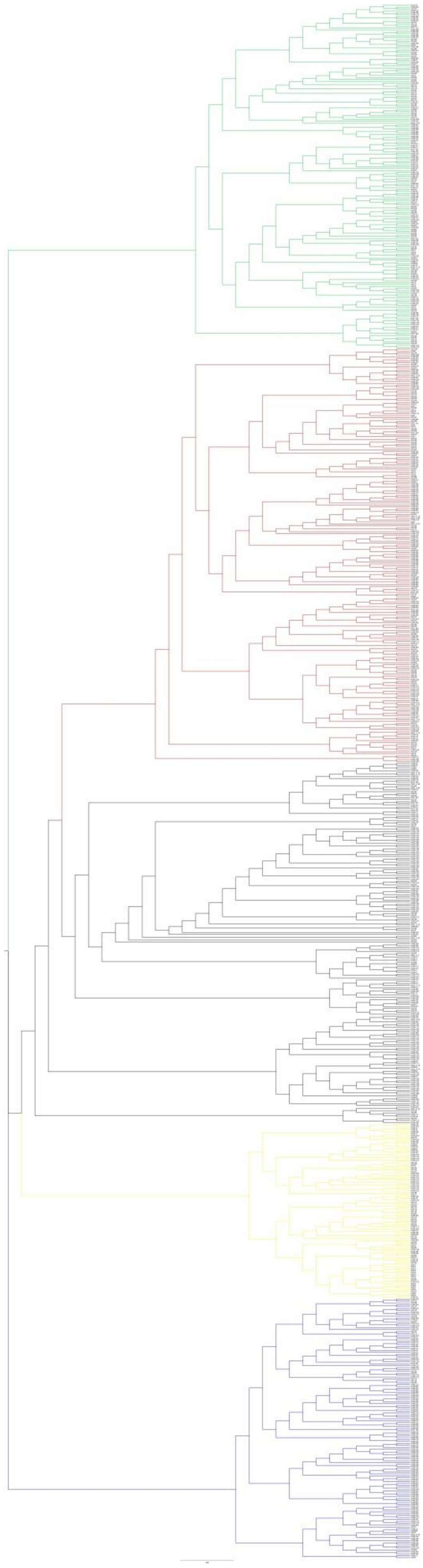

Figure S3. Neighbor-joining (NJ) tree for 818 maize inbred lines used for GWAS. The green and blue colours indicate the two small major subgroups which mainly come from CIMMYT drought tolerance population La Posta Sequia (LaPostSeq) and lowland breeding program, respectively. The red, black and orange colours indicates that the major subgroup come from broadly breeding program, including the CIMMYT lowland breeding program, physiology, pathology, and breeding programs in Africa.
