# Supplementary material for: Genome-Wide Association Study and QTL Mapping Reveal Genomic Loci Associated with Fusarium Ear Rot Resistance in Tropical Maize Germplasm
Source: G3 (Bethesda). 2016 Oct 13;6(12):3803–15. doi: 10.1534/g3.116.034561 (PMC5144952; doi:10.1534/g3.116.034561)
Supplement: Supplemental Material [file supp_g3.116.034561_FigureS4.pdf]

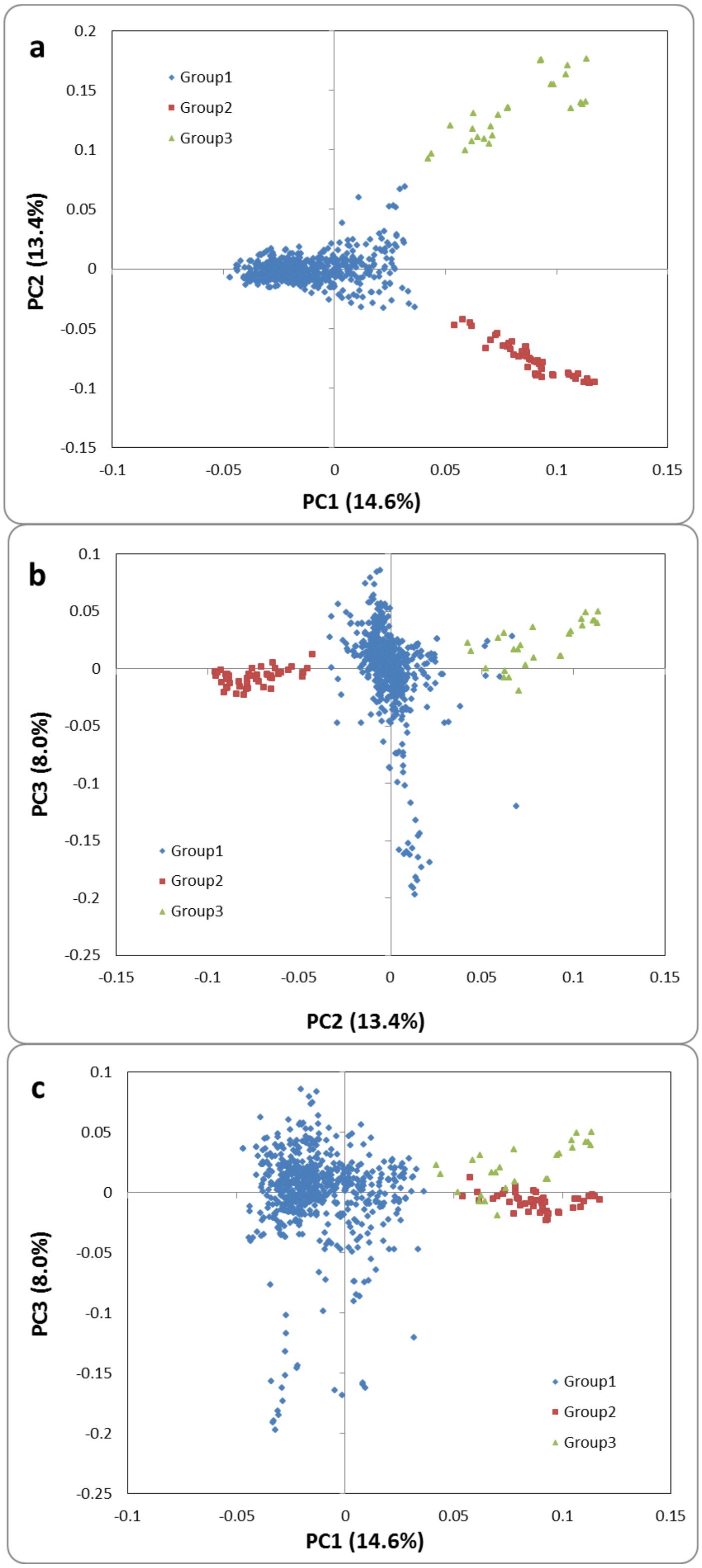

a
Group1
Group2
Group3
PC2 (13.4%)
PC1 (14.6%)
b
PC3 (8.0%)
PC2 (13.4%)
c
PC3 (8.0%)
PC1 (14.6%)

Figure S4. Population structure based on principal component analysis (PCA) for 818 maize inbred lines included in association mapping (a: PC1 vs. PC2; b: PC2 vs PC3; c: PC1 vs PC3). The lines was clustered into 3 subgroups using k-mean method, Group 2 (red) and 3 (green) are composed of germplasm derived from LaPostaSeq population and some lines from the lowland breeding program, respectively. Group 1 (blue) is made up of lines derived from CIMMYT's breeding programs in Africa, the lowland breeding program, physiology, and pathology programs.
