# Supplementary material for: Genome-Wide Association Study and QTL Mapping Reveal Genomic Loci Associated with Fusarium Ear Rot Resistance in Tropical Maize Germplasm
Source: G3 (Bethesda). 2016 Oct 13;6(12):3803–15. doi: 10.1534/g3.116.034561 (PMC5144952; doi:10.1534/g3.116.034561)
Supplement: Supplemental Material [file supp_g3.116.034561_FigureS6.pdf]

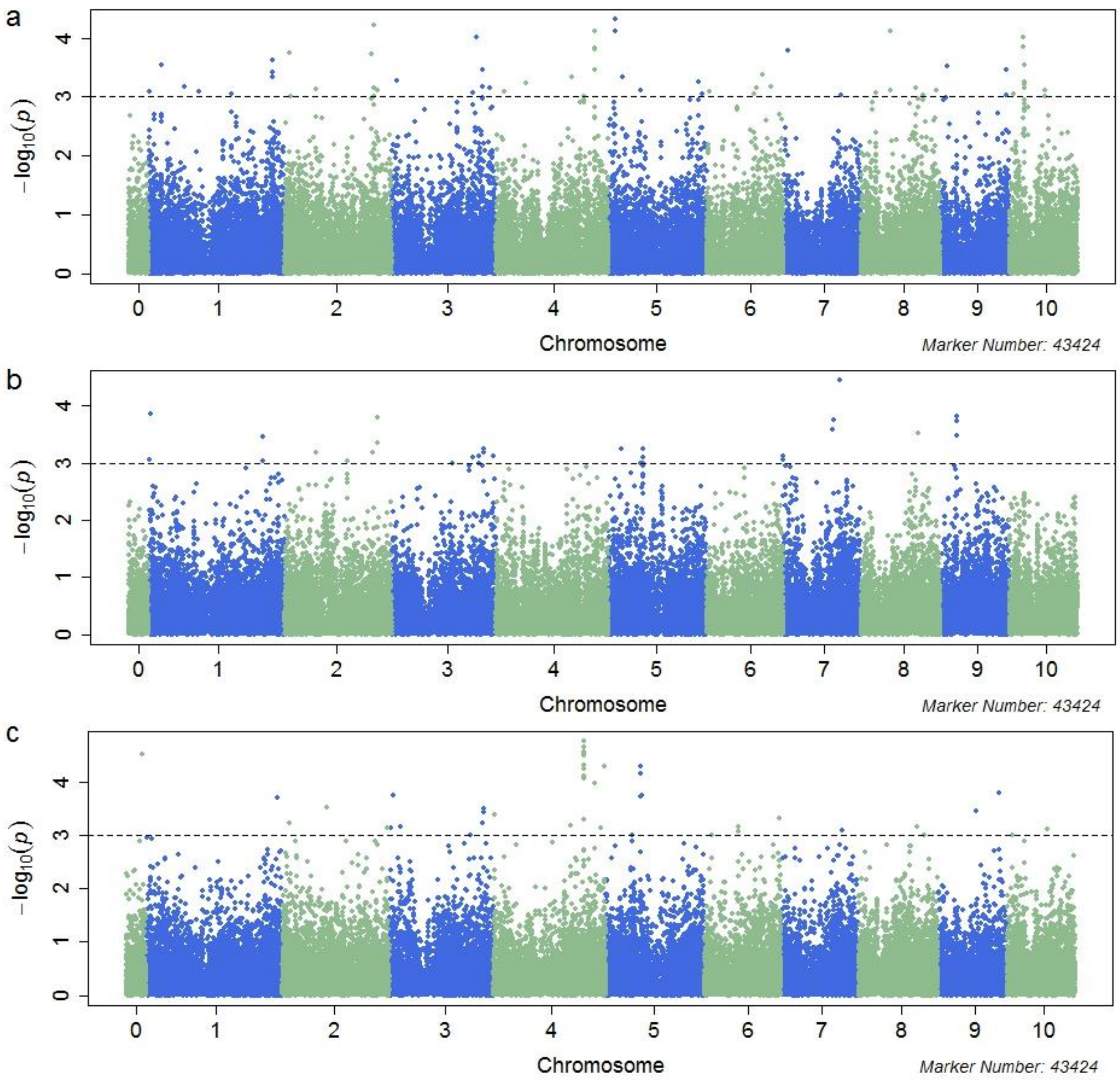


**Figure S6.** Manhattan plots of mixed linear model for *Fusarium* ear rot (FER) resistance for 818 maize inbred lines in single environment GWAS. The environments are (a) TL11, (b) AF11, and (c) AF10. The vertical axis indicates $-\log_{10}$ of *p*-value scores, and the horizontal axis indicates chromosomes and physical positions of SNPs. Plots above the horizontal dash line showed the genome-wide significance with threshold of $P = 3 \times 10^{-3}$.
