# Supplementary figures and images for: Genome-Wide Association Study and QTL Mapping Reveal Genomic Loci Associated with Fusarium Ear Rot Resistance in Tropical Maize Germplasm
Source: G3 (Bethesda). 2016 Oct 13;6(12):3803–15. doi: 10.1534/g3.116.034561 (PMC5144952; doi:10.1534/g3.116.034561)

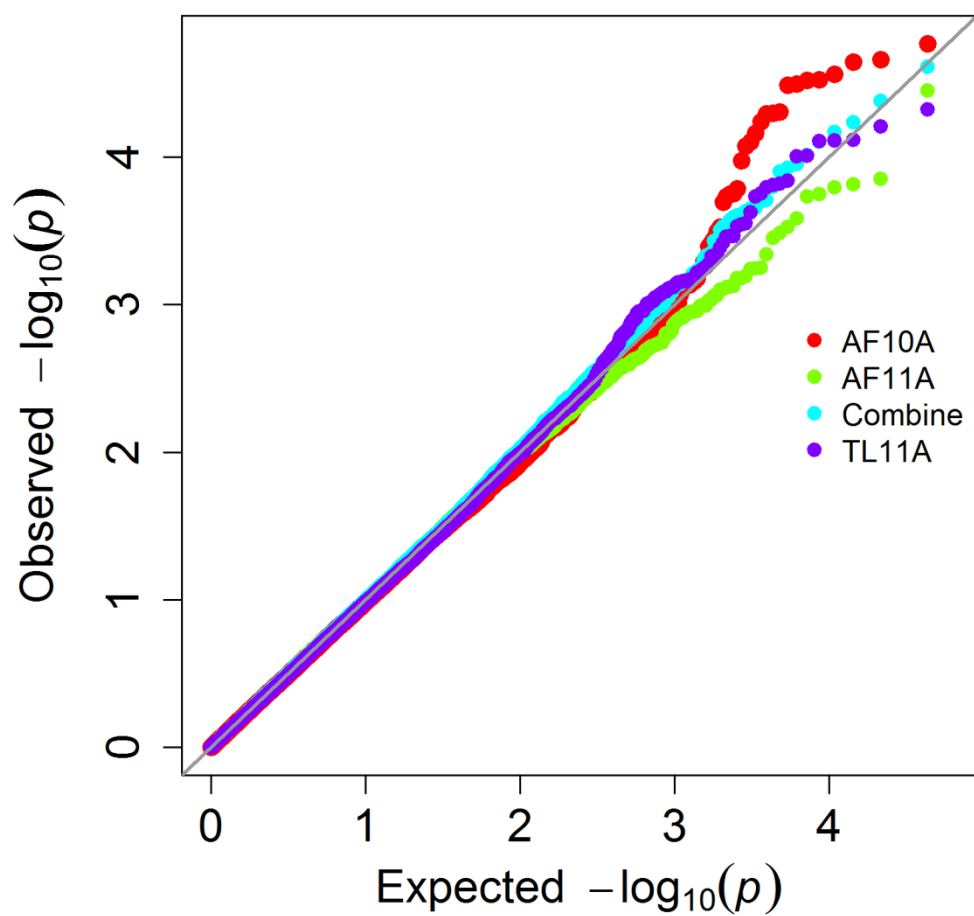

**Figure S7.** QQ plots resulting from single markers GWAS for the GWAS panel.

Supplement: Supplemental Material [file supp_g3.116.034561_FigureS7.pdf]
